# Circ_0006646 Promotes the Progression of Osteoarthritis via Upregulating CDH11 Expression in an IGF2BP2‐Dependent Manner

**DOI:** 10.1002/kjm2.70031

**Published:** 2025-05-19

**Authors:** Ming‐Yu Hua, Guo‐Liang Wang, Wen‐Hao Duan, Xiao‐Heng Tang

**Affiliations:** ^1^ Department of Orthopaedics Tangdu Hospital, Fourth Military Medical University Xi'an Shaanxi China; ^2^ Department of Orthopaedics Xi'an Medical University Xi'an Shaanxi China

**Keywords:** CDH11, chondrocyte, circ_0006646, IGF2BP2, osteoarthritis

## Abstract

Osteoarthritis (OA) is a common degenerative osteoarthropathy with an unclear pathogenesis. Circular RNA (circRNA) has been reported to be associated with OA progression. This study aims to explore the role and potential mechanism of hsa_circ_0006646 in OA. Interleukin‐1β (IL‐1β)‐induced human chondrocytes were used as the cell model of OA. RT‐qPCR and western blotting were used to detect the expression of circ_0006646, IGF2BP2, and cadherin 11 (CDH11). Cell counting kit‐8 assay, 5‐ethynyl‐2′‐deoxyuridine assay, and flow cytometry were performed to assess chondrocyte cell proliferation and apoptosis, respectively. Western blot assay was performed to determine the levels of proliferation‐related proteins, apoptosis‐related proteins, and extracellular matrix (ECM) proteins. RNA immunoprecipitation (RIP) assay was performed to verify the interaction between IGF2BP2 and circ_0006646 or CDH11. In OA patients and IL‐1β‐stimulated chondrocytes, circ_0006646 and CDH11 were upregulated. IL‐1β suppressed proliferation and induced apoptosis, inflammation, and ECM degradation in chondrocytes, and circ_0006646 knockdown protected chondrocytes from IL‐1β‐induced damage. IGF2BP2 was proved to interact with both circ_0006646 and CDH11. The overexpression of IGF2BP2 or CDH11 enhanced IL‐1β‐induced apoptosis, inflammation, and ECM degradation in chondrocytes. Moreover, circ_0006646 absence‐mediated effects in IL‐1β‐treated chondrocytes could be largely overturned by the overexpression of IGF2BP2 or CDH11. In conclusion, circ_0006646 knockdown protected chondrocytes from IL‐1β‐induced injury by regulating CDH11 in an IGF2BP2‐dependent manner, suggesting a novel potential target for OA treatment.

## 1 Introduction

Osteoarthritis (OA) is a widespread age‐related degenerative disease characterized by chronic inflammation, progressive cartilage destruction, and subchondral bone sclerosis [1]. Study in 2019 shows that the prevalent cases of OA have increased by 113% since 1990, and about 528 million people worldwide were living with OA [2, 3]. Although the exact pathogenesis of OA is still unclear, dysregulated programmed cell death, sustained inflammation, and extracellular matrix (ECM) destruction have been identified as important risk factors for disease progression [4, 5]. Patients with OA are forced to undergo prolonged periods of pain and joint dysfunction, eventually reaching an advanced stage and relying on surgery for relief [6]. Therefore, it is important to study the pathogenesis of OA in depth to find effective therapeutic targets to halt or reverse its progression.

Circular RNAs (circRNAs) are a type of non‐coding competitive endogenous RNAs discovered in recent years, with closed covalently continuous loops without 5′‐3′ structures and Poly A tails [7, 8]. With the development of technology, many circRNAs have been discovered and identified in human cells, arousing great interest among researchers [9]. CircRNAs are reported to have multiple biological functions in disease regulation [10]. For example, circRNA‐PTPRA could inhibit atherosclerosis progression [11]. Circ_0008934 stimulated osteosarcoma growth and metastasis through the regulation of miR‐145‐5p/E2F3 [12]. RNA sequencing by Yang et al. identified a total of 27,318 circRNAs in chondrocytes, among which hsa_circ_0006646 exhibited significant upregulation after either IL‐β or H_2_O_2_ treatment [13]. This study will investigate hsa_circ_0006646 in chondrocytes to explore its role and mechanism in OA progression.

## 2 Materials and Methods

### 2.1 Articular Cartilage Tissues

OA cartilage tissues and normal cartilage tissues were isolated from the knee joints of 30 patients with OA and 21 patients with amputation trauma at Tangdu Hospital, Fourth Military Medical University. Patients' inclusion criteria: (1) newly diagnosed OA cases, (2) no therapy was initiated prior to admission, and (3) patients were willing to participate in the study. Recurrent cases and patients with other clinical disorders, inflammation, and infection were excluded. The clinical characteristics of the patients with OA and the Controls are shown in Table S1. The study was approved by the Ethics Committee of Tangdu Hospital, Fourth Military Medical University (approved number: 202309‐16‐KYB‐08‐XY‐01). Tissue samples were processed further after the acquirement of informed consent from patients.

### 2.2 Chondrocyte Isolation and Culture

Knee cartilage specimens was cut into small pieces under an aseptic environment. Then, collagenase II was added at a 2:1 volume ration, digested at 37°C, filtered with a cell screen, and centrifuged at 800 rpm for 6 min. After removing the supernatant, chondrocytes were resuspended in DMEM culture medium (Gibco, Carlsbad, CA, USA) containing 15% FBS, and maintained at 37°C in a humidified 5% CO^2^ atmosphere. Chondrocytes at the 2nd‐3rd generation were used for functional analyses.

### 2.3 Cell Treatment and Cell Transfection

Chondrocytes were stimulated for 24 h with 10 ng/mL IL‐1β at 37°C to simulate the OA cell model. Short hairpin (sh) RNAs of circ_0006646 (sh‐circ_0006646) or IGF2BP2 (sh‐IGF2BP2), non‐targeting control shRNA (sh‐NC), circ_0006646/cadherin‐11 (CDH11)/IGF2BP2 expressing plasmids in pcDNA vector (oe‐circ_0006646, CDH11 or oe‐IGF2BP2), and empty plasmids (vector) were provided by Genepharma (Shanghai, China) and Ribobio (Guangzhou, China). Cell transfection was performed using Lipofectamine 2000 (Invitrogen, Carlsbad, CA, USA).

### 2.4 RT‐qPCR

Total RNA was extracted by Trizol (Invitrogen) and then reverse‐transcribed into cDNAs using a High‐Capacity cDNA Reverse Transcription Kit (Applied Biosystems, Foster City, CA, USA), and PCR was performed using SYBR Premix Ex Taq II (TaKaRa) with 7500 Real‐Time PCR System (Applied Biosystems, Foster City, CA, USA). The 2^−ΔΔCt^ method was used for relative quantification with GAPDH or U6 as the housekeeping gene. The primer sequences are listed in Table 1.

**TABLE 1:** Primer sequences used for qPCR.

Name		Primers for PCR (5′–3′)
hsa_circ_0006646	Forward	CCCACCAGAGGAGTGGAAAAT
	Reverse	GGTGTGTGATTCAAGTTGGGG
PTK2	Forward	TGGGCGGAAAGAAATCCTGC
	Reverse	GGCTTGACACCCTCGTTGTA
CDH11	Forward	GGGTGGGGAAGAAGACACAG
	Reverse	AGCCCAGGTCTAGGCATGTA
GAPDH	Forward	ACAACTTTGGTATCGTGGAAGG
	Reverse	GCCATCACGCCACAGTTTC
U6	Forward	CTCGCTTCGGCAGCACA
	Reverse	AACGCTTCACGAATTTGCGT

### 2.5 CircRNA Stability Analysis

The chondrocytes were inoculated in 6‐well plates and cultured overnight. 2 μg of total RNA was digested with 2 U/μg RNase R (Glpbio, Montclair, CA, USA) for 1 h and then detected by RT‐qPCR. Total cellular RNA was extracted at 0, 8, 16, and 24 h after treatment with 2 mg/mL Actinomycin D (Glpbio), respectively, and then detected by RT‐qPCR.

### 2.6 Nucleoplasmic Separation Experiment

The cells were collected, and the RNA in the cytoplasm and nucleus was isolated and extracted using the PARIS Kit (Invitrogen). The subcellular distribution of circ_0006646 was analyzed, and U6 or GAPDH served as the internal reference for nuclear or cytoplasmic fraction, respectively.

### 2.7 CCK‐8 Assay

The chondrocytes were inoculated into 96‐well plates at 3 × 10^4^ per well. The cells were incubated for 0, 1, 2, and 3 d. Then, each well was added with 10 μL of CCK‐8 solution (Glpbio). Following a 1.5 h incubation at 37°C, cell proliferation viability was assessed by measuring the A570 nm value with a microplate reader.

### 2.8 5‐Ethynyl‐2′‐Deoxyuridine (EdU) Assay

Chondrocytes were inoculated into 96‐well plates, and 100 μL EdU medium (50 μM, Ribobio) was added to each well for 2 h. Then, cells were fixed with 4% paraformaldehyde for 0.5 h, and permeabilized with 0.5% Triton X‐100 for 10 min. Next, 100 μL 1 × Merge staining solution was added and incubated for 30 min, followed by 5 μL Hoechst staining for 5 min, sheltering from light. Ten fields of view were randomly photographed under a fluorescent microscope.

### 2.9 Western Blot

The total cellular protein was extracted routinely and separated by SDS‐PAGE at appropriate concentrations, then transferred to the PVDF membrane. The membrane was blocked with 5% BSA for 1 h, incubated with primary antibodies at 4°C overnight, followed by incubation with the corresponding secondary antibody (1:50000 dilution, ab205718; Abcam, Cambridge, MA, USA) at room temperature for 1 h. Protein bands were detected in the gel imager by adding an ECL working solution (Share, Shanghai, China). The primary antibodies used in the experiments are shown in Table 2.

**TABLE 2:** Primary antibodies.

Protein name	Dilution ratio	Product code	Company
PCNA	1:1000	ab92552	Abcam
Bcl‐2	1:1000	ab32124	Abcam
Bax	1:1000	ab32503	Abcam
Cleaved PARP1	1:1000	ab32064	Abcam
Collagen II	1:1000	ab188570	Abcam
Aggrecan	1:1000	ab3778	Abcam
MMP13	1:1000	ab39012	Abcam
ADAMTS5	1:1000	ab41037	Abcam
CDH11	1:1000	ab151302	Abcam
GAPDH	1:1000	ab9485	Abcam

### 2.10 Flow Cytometry

Each group of cells was incubated for 48 h, rinsed twice with pre‐cooled PBS, and mixed with 500 μL of binding buffer. Then, a total of 10 μL of Annexin V‐FITC and 5 μL of PI (BD Biosciences, Franklin Lakes, NJ, USA) were added to incubate with the cells for 10 min with protection from light. The cell apoptotic rate was analyzed by a FACSCalibur Flow Cytometer (BD Biosciences).

### 2.11 ELISA Detection

The cell culture supernatant of each group was collected, and the supernatant was added to the ELISA plate (100 μL per well) and incubated at 37°C for 1.5 h. The levels of TNF‐α and IL‐1β were detected using corresponding ELISA kits (R&D Systems, Shanghai, China).

### 2.12 RNA Immunoprecipitation (RIP) Assay

Chondrocytes transfected with sh‐circ_0006646, oe‐circ_0006646, or respective controls were lysed in cell lysate, and IGF2BP2 antibody (Abcam) or IgG antibody (Abcam) was incubated with magnetic beads for 2 h. Then the cell lysate was incubated with magnetic beads (Millipore) overnight. After washing, the proteins in the RNA‐protein complex were digested. Finally, the RNA was purified and detected by RT‐qPCR.

### 2.13 Statistical Analysis

All data are expressed as mean ± standard deviation. GraphPad Prism 7.0 was applied to analyze two independent data sets by Student's *t*‐test in two groups and by one‐way ANOVA in multiple groups. GraphPad Prism 7.0 was also used to plot the Figures. Statistical significance was standardized as *p* < 0.05.

## 3 Results

### 3.1 Circ_0006646 Expression Was Upregulated in OA

**FIGURE 1:**
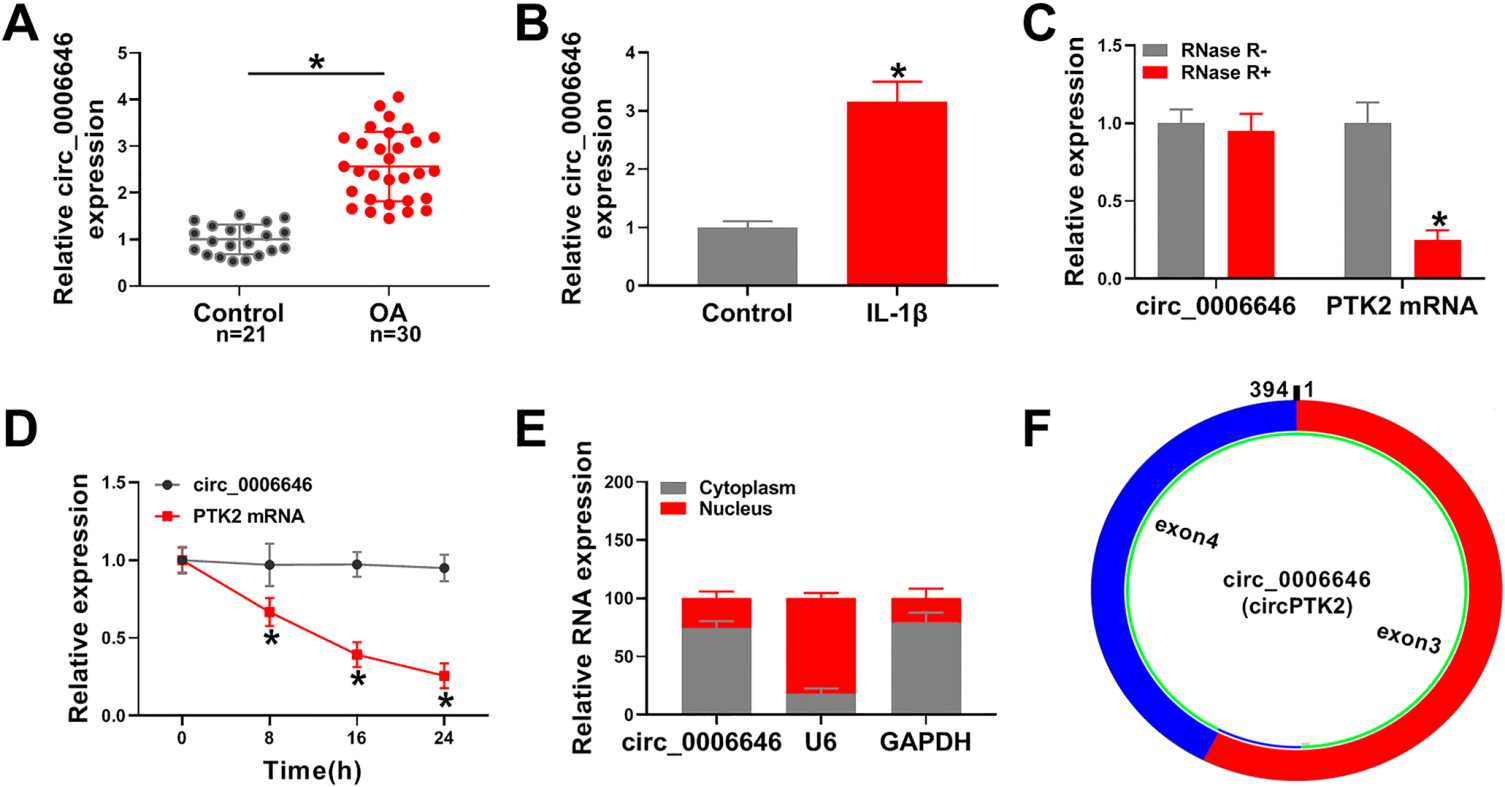
Circ_0006646 expression was upregulated in OA. (A) RT‐qPCR was conducted to detect circ_0006646 expression in OA tissues. (B) Expression of circ_0006646 was detected by RT‐qPCR after IL‐1β treatment of chondrocytes. (C and D) RT‐qPCR assay was conducted to analyze the effects of RNase R and actinomycin D on circ_0006646 and linear PTK2. (E) Nucleoplasmic separation assay was performed to assess the localization of circ_0006646 in chondrocytes. (F) The circular structure of circ_0006646 was shown. *p < 0.05.

First, RT‐qPCR was performed to detect the expression level of circ_0006646. There was a significant elevation of circ_0006646 expression in articular tissues of OA patients compared with normal articular tissues (Figure 1A). Patients were grouped as a high circ_0006646 group and a low circ_0006646 group based on the median level. There was no correlation between circ_0006646 expression and disease severity (Table S2). Then we used IL‐1β‐treated chondrocytes to simulate an osteoarthritic cell model. Chondrocytes were treated with increasing doses of IL‐1β (0, 1, 5, 10, or 20 ng/mL) for 24 h. Figure S1A showed that circ_0006646 expression was increased at 5, 10, and 20 ng/mL. Then chondrocytes were treated with 10 ng/mL for 0, 6, 12, 24, or 48 h. Circ_0006646 levels were increased at 12, 24, and 48 h (Figure S1B). Thus, chondrocytes were treated with 10 ng/mL for 24 h to induce the osteoarthritic cell model. The expression of circ_0006646 was also significantly increased in IL‐1β‐treated chondrocytes compared to untreated chondrocytes (Figure 1B). We used RNase R and actinomycin D to confirm the circular structure of circ_0006646. The results showed that RNase R only degraded PTK2 mRNA and actinomycin D only affected the expression of PTK2 mRNA, thus confirming the circular structure of circ_0006646 (Figure 1C–1D). We found that circ_0006646 mainly existed in the cytoplasm of chondrocytes by nucleoplasmic separation assay (Figure 1E). Circ_0006646 was a circRNA molecule of 394 bp in length (Figure 1F). Overall, the results indicated that circ_0006646 was highly expressed in OA.

### 3.2 Circ_0006646 Knockdown Promoted the Proliferation and Inhibited the Apoptosis and ECM Degradation in IL‐1β‐Treated Chondrocytes

**FIGURE 2:**
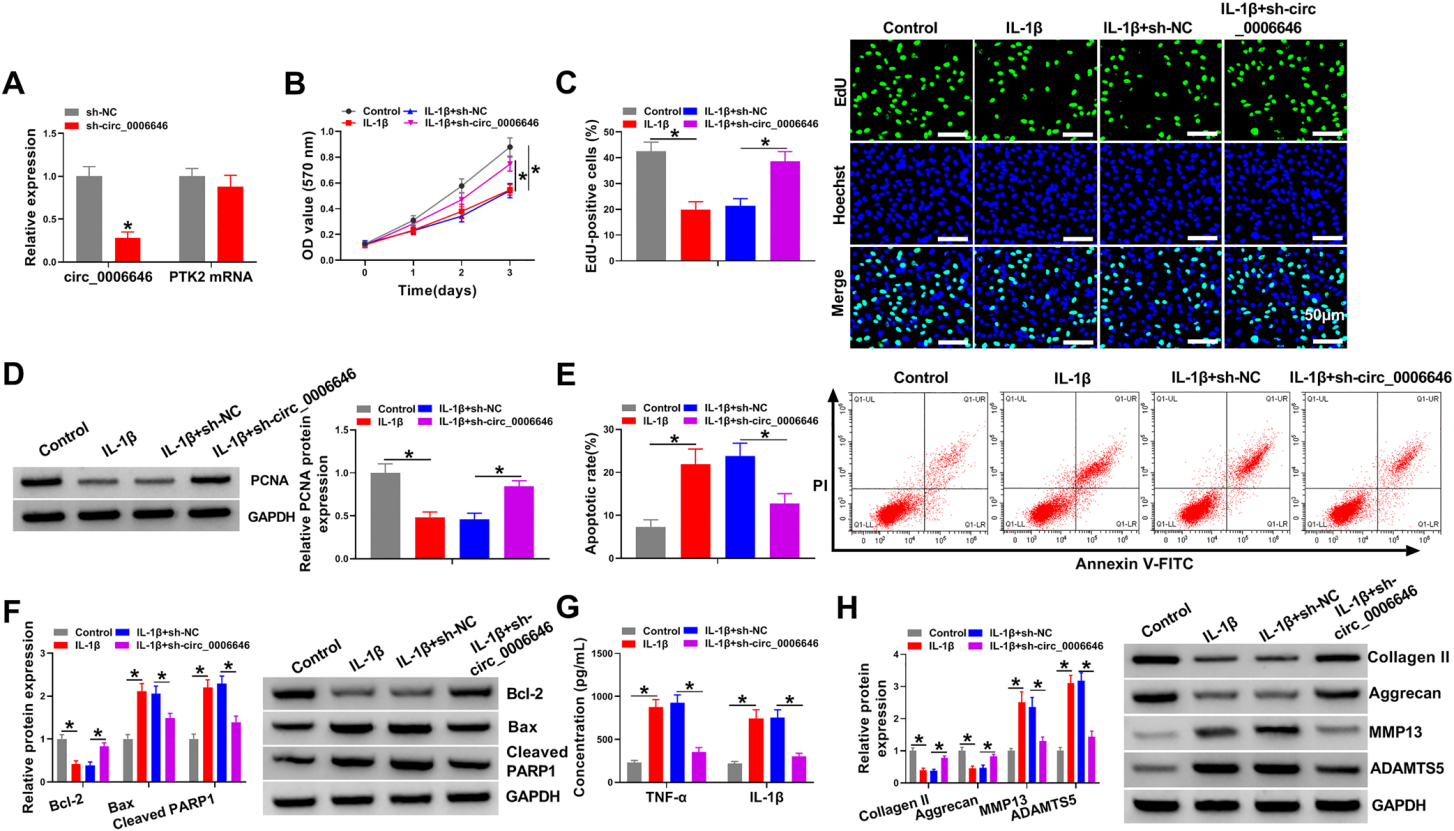
Circ_0006646 regulated cell proliferation, apoptosis, inflammation, and ECM degradation in IL‐1β‐treated chondrocytes. (A) RT‐qPCR was carried out to measure the knockdown efficiency of sh‐circ_0006646 in chondrocytes. (B–H) Chondrocytes were divided into four groups: Control, IL‐1β, IL‐1β + sh‐NC, and IL‐1β + sh‐circ_0006646. (B and C) CCK‐8 and EdU assays were performed to detect cell proliferation. (D) Western blot was conducted to detect the protein expression of PCNA. (E) Flow cytometry was conducted to detect cell apoptosis. (F) Western blot was conducted to detect the expression of apoptosis proteins Bcl‐2, Bax, and Cleaved PARP. (G) ELISA was carried out to detect the levels of inflammatory factors TNF‐α and IL‐1β. (H) Western blot was implemented to detect the expression of ECM proteins (Collagen II, Aggrecan, MMP13, and ADAMTS5). *p < 0.05.

IL‐1β‐treated chondrocytes were transfected with sh‐circ_0006646 or sh‐NC to determine the role of circ_0006646 in OA progression. RT‐qPCR results showed that sh‐circ_0006646 significantly reduced circ_0006646 expression and had no effect on PTK2 mRNA expression (Figure 2A). CCK‐8 and EdU assays showed that IL‐1β treatment of chondrocytes significantly reduced chondrocyte proliferation, and circ_0006646 knockdown reversed this reduction (Figure 2B–2C). Expression of the proliferation protein PCNA in chondrocytes was inhibited by IL‐1β, and was increased after circ_0006646 knockdown (Figure 2D). Flow cytometry analysis showed that IL‐1β stimulated chondrocyte apoptosis, while circ_0006646 knockdown attenuated this stimulation (Figure 2E). Bcl‐2 expression in chondrocytes was reduced after IL‐1β treatment, and Bax and Cleaved PARP1 were increased after IL‐1β treatment, and these effects were rescued by sh‐circ_0006646 transfection (Figure 2F). Cytokine TNF‐α and IL‐1β levels in chondrocytes were increased after IL‐1β treatment, while knockdown of circ_0006646 in IL‐1β‐treated chondrocytes decreased TNF‐α and IL‐1β levels (Figure 2G). The expression of ECM proteins Collagen II and Aggrecan was decreased, and the expression of MMP13 and ADAMTS5 was increased in IL‐1β‐treated chondrocytes, and these effects were largely overturned by circ_0006646 knockdown (Figure 2H). Overall, knockdown of circ_0006646 suppressed IL‐1β‐induced dysfunction in chondrocytes by regulating cell proliferation, apoptosis, inflammation, and ECM degradation.

### 3.3 CDH11 Expression Was Upregulated in OA and IL‐1β‐Induced Chondrocytes

**FIGURE 3:**
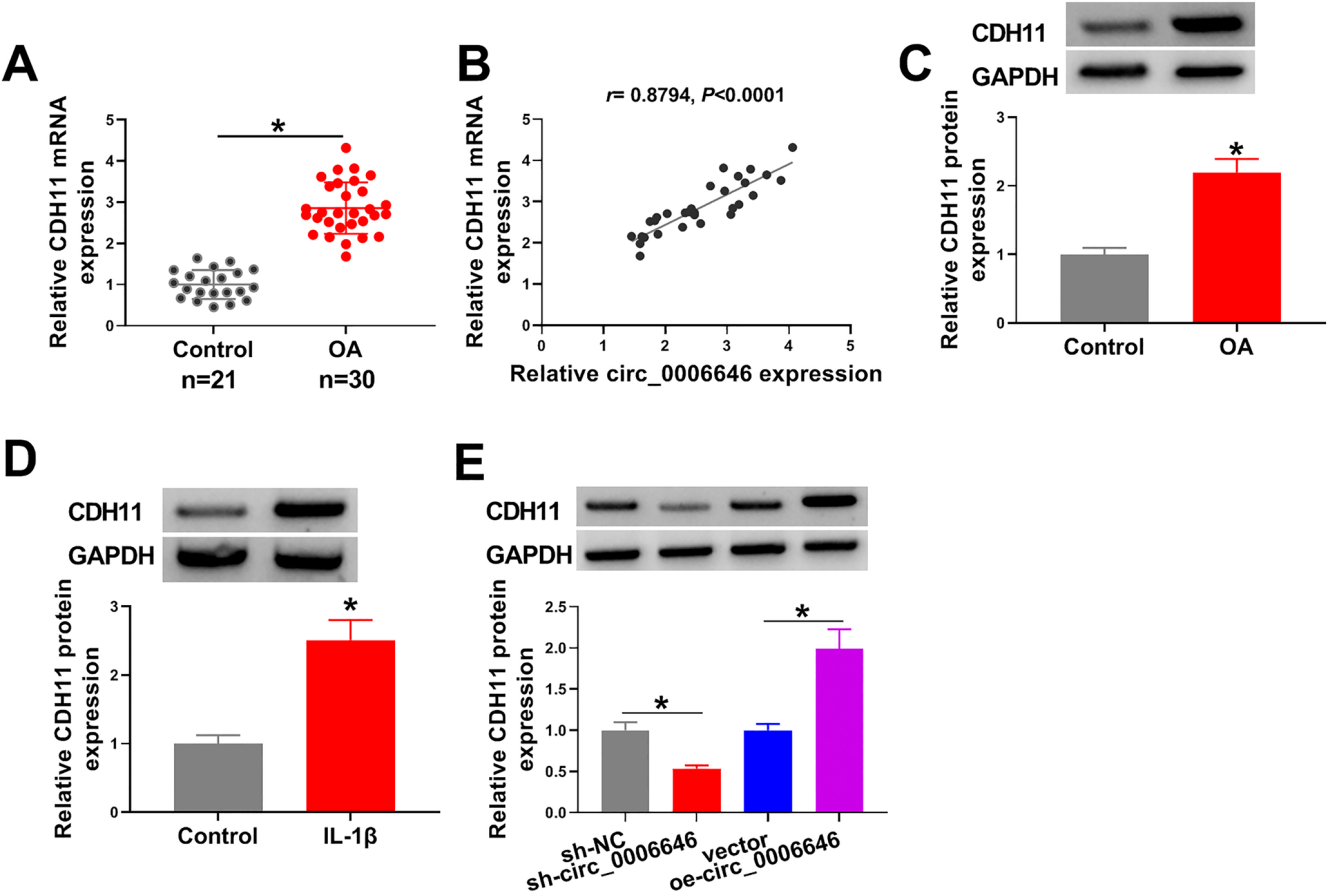
CDH11 expression was upregulated in OA and IL‐1β‐induced chondrocytes. (A) RT‐qPCR was conducted to detect CDH11 mRNA expression in OA tissues. (B) The correlation analysis between CDH11 mRNA and circ_0006646 expression in OA tissues. (C and D) Western blotting analysis was conducted to detect CDH11 protein expression in OA tissues and IL‐1β‐treated chondrocytes. (E) Western blotting analysis for CDH11 protein expression in IL‐1β‐treated chondrocytes after circ_0006646 overexpression or down‐regulation. *p < 0.05.

In OA tissues, we also found CDH11 mRNA expression was significantly increased and positively correlated with circ_0006646 expression (Figure 3A–3B). Also, CDH11 protein expression was increased in OA tissues (Figure 3C). Moreover, IL‐1β also markedly elevated CDH11 expression in chondrocytes (Figure 3D). Subsequently, we found that CDH11 expression was decreased by circ_0006646 silencing, but was increased by circ_0006646 upregulation in IL‐1β‐induced chondrocytes (Figure 3E), further suggesting that circ_0006646 positively regulated CDH11 expression in chondrocytes.

### 3.4 Circ_0006646 Regulated the Proliferation, Apoptosis, Inflammation, and ECM Degradation in IL‐1β‐Treated Chondrocytes by CDH11

**FIGURE 4:**
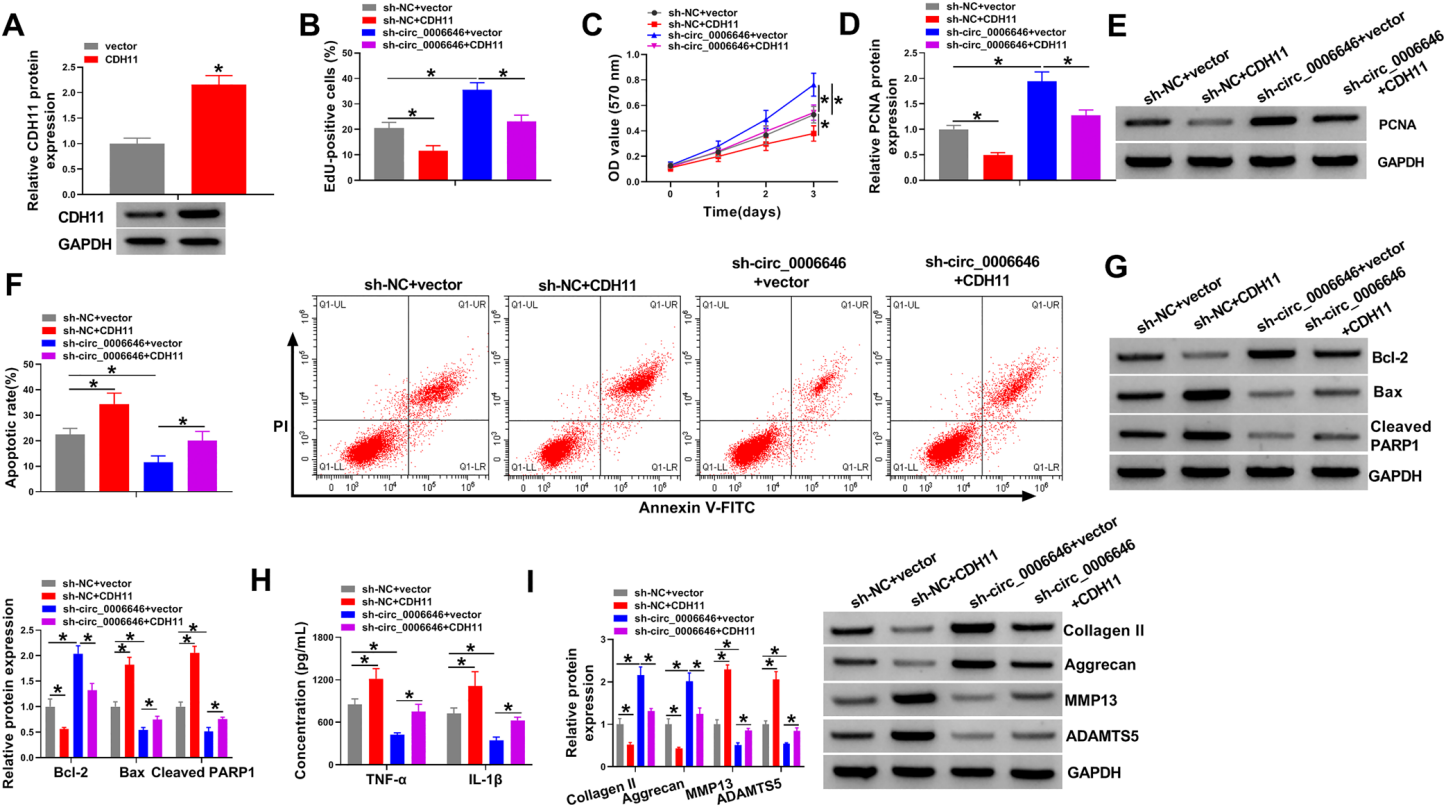
Circ_0006646 regulated the proliferation, apoptosis, inflammation, and ECM degradation in IL‐1β‐treated chondrocytes by CDH11. (A) Western blotting was carried out to measure the expression of CDH11 in chondrocytes with CDH11 transfection. (B and C) CCK‐8 and EdU assays were performed to detect cell proliferation. (D and E) Western blot was conducted to detect the protein expression of PCNA. (F) Flow cytometry was conducted to detect cell apoptosis. (G) Western blot was conducted to detect the expression of apoptosis proteins Bcl‐2, Bax, and Cleaved PARP. (H) ELISA was carried out to detect the levels of inflammatory factors TNF‐α and IL‐1β. (I) Western blot was implemented to detect the expression of ECM proteins (Collagen II, Aggrecan, MMP13, and ADAMTS5). *p < 0.05.

Western blotting analysis showed that CDH11 vector introduction markedly elevated CDH11 expression in chondrocytes (Figure 4A). Thereafter, chondrocytes were transfected with sh‐NC and CDH11, sh‐circ_0006646 alone, or sh‐circ_0006646 and CDH11, and then exposed to IL‐1β. Functionally, CCK‐8 and EdU experiments showed that CDH11 overexpression suppressed the proliferation of IL‐1β‐treated chondrocytes, sh‐circ_0006646 promoted the proliferation of IL‐1β‐treated chondrocytes, and the effect of CDH11 and sh‐circ_0006646 on proliferation would cancel each other out after cotransfection (Figure 4B,C). CDH11 reduced PCNA expression in IL‐1β‐treated chondrocytes, and the increased expression of PCNA protein after circ_0006646 knockdown was reversed by CDH11 overexpression (Figure 4D,E). CDH11 promoted the apoptosis of IL‐1β‐treated chondrocytes and restored the inhibition of apoptosis by circ_0006646 knockdown (Figure 4F). In contrast to sh‐circ_0006646 transfection, CDH11 inhibited Bcl‐2 protein expression and promoted Bax and Cleaved PARP1 protein expression. And sh‐circ_0006646 cotransfection with CDH11 resulted in little difference in the expression of these three proteins from that before transfection (Figure 4G). In addition, CDH11 was able to increase the levels of TNF‐α and IL‐1β in IL‐1β‐treated chondrocytes, and CDH11 was also able to restore the reduced levels of TNF‐α and IL‐1β caused by circ_0006646 knockdown (Figure 4H). CDH11 elevation reduced the expression of Collagen II and Aggrecan and upregulated the expression of MMP13 and ADAMTS5 in circ_0006646‐silenced chondrocytes (Figure 4I). The above results showed that circ_0006646 regulated CDH11 expression to modulate chondrocyte proliferation, apoptosis, inflammation, and ECM degradation.

### 3.5 Circ_0006646 Elevated CDH11 Expression in Chondrocytes in an IGF2BP2‐Dependent Manner

**FIGURE 5:**
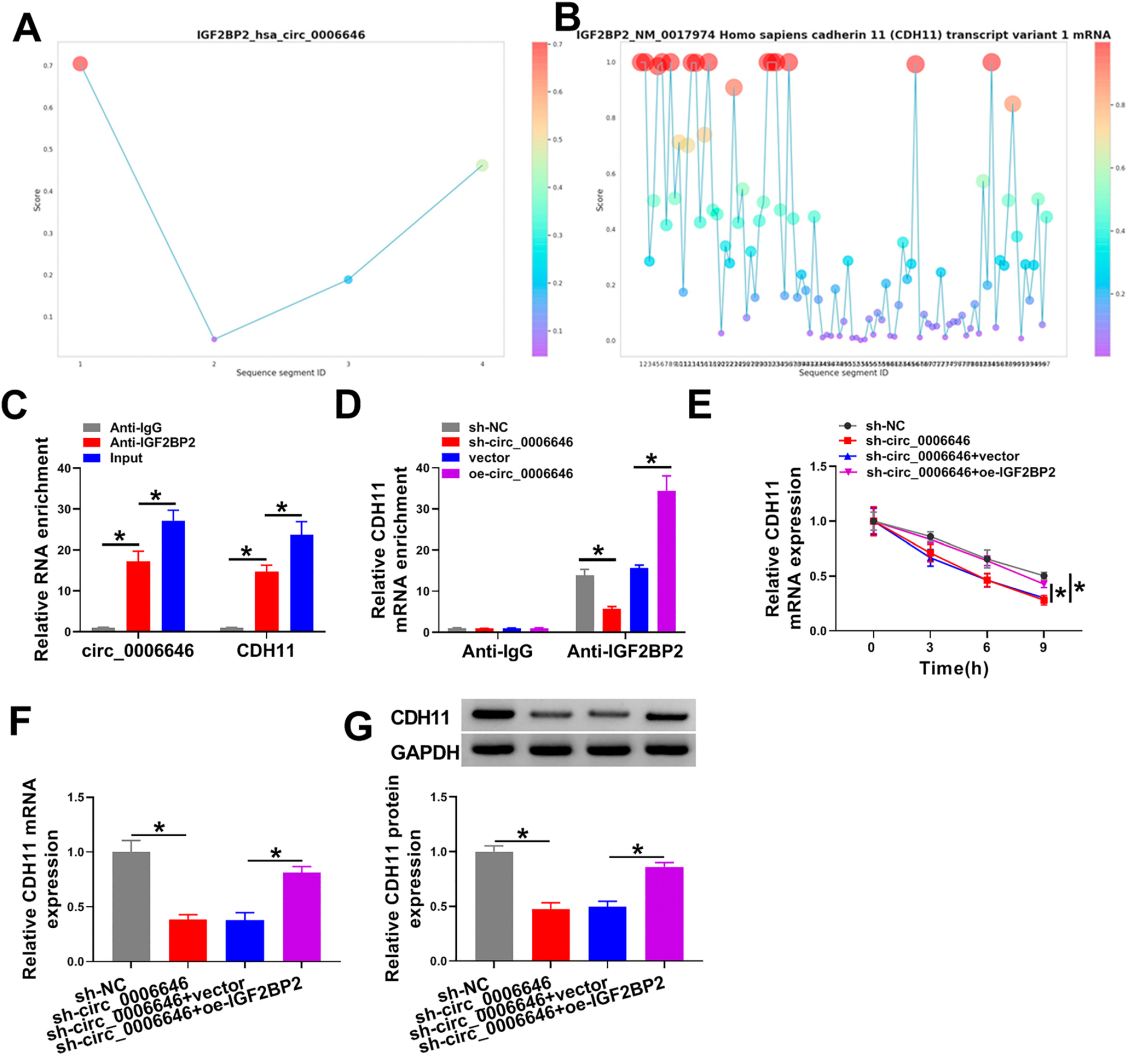
Circ_0006646 elevated CDH11 expression in chondrocytes in an IGF2BP2‐dependent manner. (A and B) RBPsuit database predicted that IGF2BP2 may interact with circ_0006646 and CDH11. (C and D) RIP assay was used to confirm the interaction between IGF2BP2 and CDH11 or circ_0006646. (E) The effects of circ_0006646 and IGF2BP2 on the stabilization of CDH11 were detected by Actinomycin D treatment. (F and G) The effects of circ_0006646 and IGF2BP2 on the expression of CDH11 were detected by RT‐qPCR and western blotting. *p < 0.05.

Through the RBPsuit database, we found IGF2BP2 may interact with circ_0006646 and CDH11 (Figure 5A,B). Therefore, we explored whether circ_0006646 affected CDH11 expression via IGF2BP2. The RIP assay showed that CDH11 and circ_0006646 were markedly pulled down by IGF2BP2 (Figure 5C), and circ_0006646 overexpression increased the enrichment of CDH11 on IGF2BP2, while circ_0006646 silencing reduced the enrichment of CDH11 on IGF2BP2 (Figure 5D). Actinomycin D treatment suggested that circ_0006646 silencing reduced the stabilization of CDH11 mRNA, which could be rescued by IGF2BP2 overexpression (Figure 5E). Moreover, circ_0006646 silencing led to the decrease of CDH11 expression, and the reduction of CDH11 was restored by IGF2BP2 overexpression (Figure 5F,G). In conclusion, circ_0006646 stabilized CDH11 expression by interacting with IGF2BP2.

### 3.6 Circ_0006646 Regulated the Proliferation, Apoptosis, Inflammation, and ECM Degradation in IL‐1β‐Treated Chondrocytes by IGF2BP2

Then, the effects of IGF2BP2 on chondrocyte dysfunction were investigated. Western blotting analysis showed that sh‐IGF2BP2 markedly reduced IGF2BP2 expression in chondrocytes (Figure S2A). Functionally, IGF2BP2 silencing abolished IL‐1β‐induced proliferation inhibition (Figure S2B–D), apoptosis enhancement (Figure S2E,F), increase in TNF‐α and IL‐1β levels (Figure S2) as well as MMP13 and ADAMTS5 protein levels (Figure S2H), and decrease in Collagen II and Aggrecan protein levels (Figure S2H) in chondrocytes.

**FIGURE 6:**
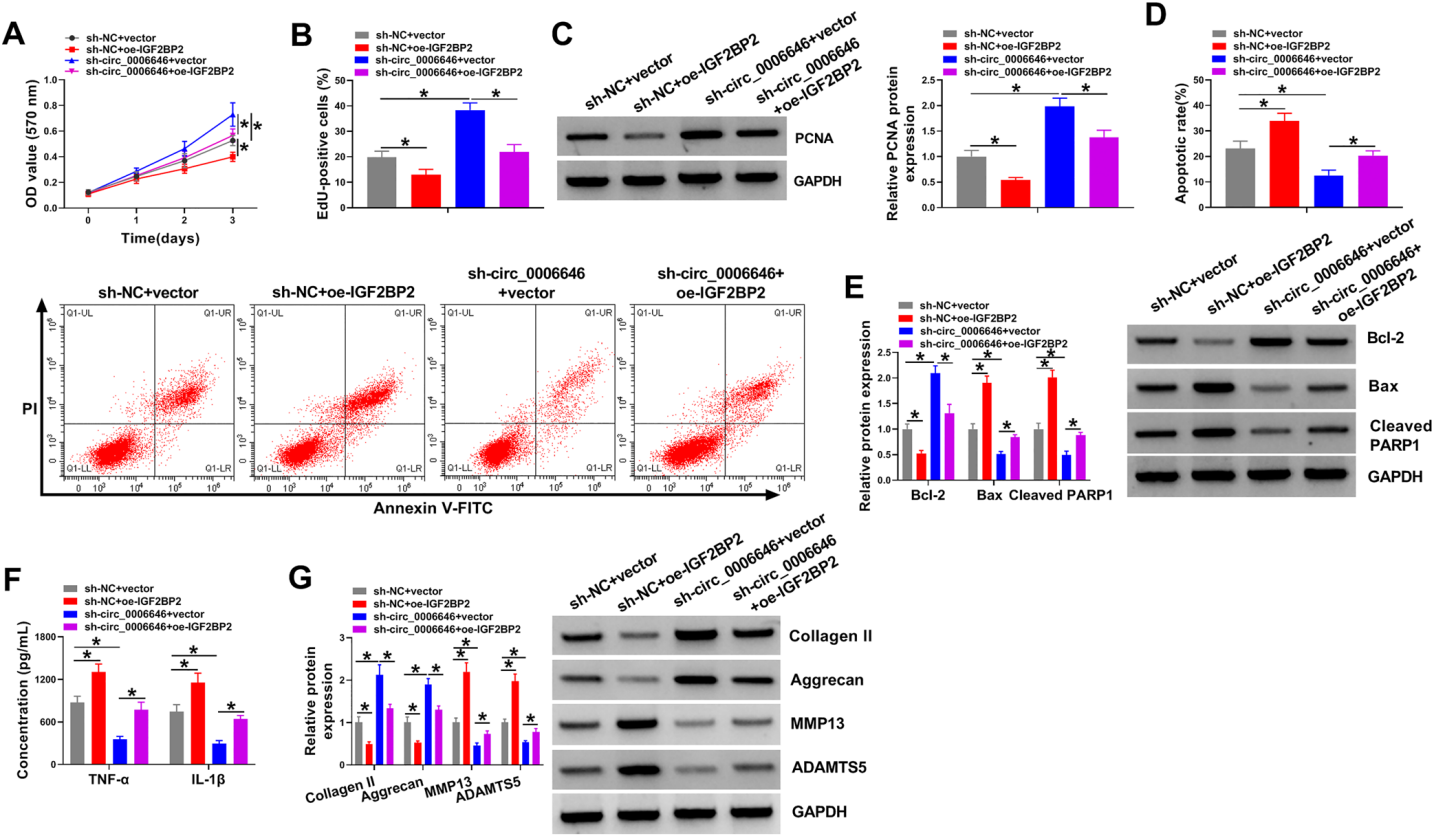
Circ_0006646 regulated the proliferation, apoptosis, inflammation, and ECM degradation in IL‐1β‐treated chondrocytes by IGF2BP2. (A and B) CCK‐8 and EdU assays were performed to detect cell proliferation. (C) Western blot was conducted to detect the protein expression of PCNA. (D) Flow cytometry was conducted to detect cell apoptosis. (E) Western blot was conducted to detect the expression of apoptosis proteins Bcl‐2, Bax, and Cleaved PARP. (F) ELISA was carried out to detect the levels of inflammatory factors TNF‐α and IL‐1β. (G) Western blot was implemented to detect the expression of ECM proteins (Collagen II, Aggrecan, MMP13, and ADAMTS5). *p < 0.05.

Next, we investigated whether IGF2BP2 mediated the effects of circ_0006646 on IL‐1β‐treated chondrocytes. Chondrocytes were transfected with sh‐NC and oe‐IGF2BP2, sh‐circ_0006646 alone, or sh‐circ_0006646 and oe‐IGF2BP2 and then exposed to IL‐1β. In IL‐1β‐treated chondrocytes, IGF2BP2 overexpression inhibited proliferation and reversed sh‐circ_0006646‐induced proliferation enhancement (Figure 6A,B). IGF2BP2 overexpression inhibited PCNA protein expression in IL‐1β‐treated chondrocytes and reversed sh‐circ_0006646‐induced increase of PCNA (Figure 6C). IGF2BP2 overexpression in IL‐1β‐treated chondrocytes promoted apoptosis, and the inhibition of cell apoptosis mediated by sh‐circ_0006646 could be rescued by IGF2BP2 overexpression (Figure 6D,E). IGF2BP2 overexpression in IL‐1β‐treated chondrocytes increased TNF‐α and IL‐1β levels and abolished sh‐circ_0006646‐induced reduction of TNF‐α and IL‐1β levels in cells (Figure 6F). Furthermore, in IL‐1β‐treated chondrocytes, Collagen II and Aggrecan proteins were decreased after increased IGF2BP2 expression, and MMP13 and ADAMTS5 proteins were increased after IGF2BP2 overexpression; besides that, the increase of Collagen II and Aggrecan proteins and decrease of MMP13 and ADAMTS5 proteins caused by sh‐circ_0006646 were abolished by IGF2BP2 overexpression (Figure 6G). Overall, circ_0006646 was involved in the regulation of chondrocyte proliferation, apoptosis, inflammation, and degradation of ECM by IGF2BP2.

## 4 Discussion

OA was a common degenerative disease involving articular cartilage destruction and subchondral bone remodeling, for which there was no specific therapy to date [14]. Chondrocytes autophagy plays a vital role in OA pathogenesis [15, 16]. Some studies had reported that IL‐1β could induce apoptosis and inflammatory responses in osteoarthritic chondrocytes, and IL‐1β could also induce chondrocyte damage [17, 18]. Therefore, in this experiment, an OA cell model was established by exposing chondrocytes to IL‐1β, and the results showed that the cell survival rate was significantly reduced in IL‐1β‐induced chondrocytes. circRNAs were unique non‐coding RNAs that were covalently linked by the 3' and 5' ends to form closed‐loop structures [19]. Recent studies have shown that circRNAs had significant effects on several key aspects of OA development and acted as potential therapeutic targets [20]. Circ‐BRWD1 knockdown attenuated IL‐1β‐induced OA progression [21]. Knockdown of hsa_circ_0037658 inhibited the progression of OA through induction of autophagy [22]. In this study, OA tissues showed high circ_0006646 expression. Circ_0006646 knockdown promoted the proliferation and ECM degradation and inhibited inflammation and apoptosis in IL‐1β‐treated chondrocytes.

Thereafter, the mechanism underlying circ_0006646 in OA progression was investigated. We demonstrated that IGF2BP2 interacted with circ_0006646 and CDH11, and circ_0006646 stabilized and upregulated CDH11 expression by IGF2BP2 in chondrocytes. IGF2BP2 is an RNA‐binding protein and has been identified that can modulate multiple biological processes through post‐transcriptionally regulating RNAs via the ribonucleoprotein complex [23, 24]. In addition, IGF2BP2 serves as a reader of RNA m6A (N6 methyladenosine) modification and is implicated in the localization, stabilization, and trafficking of target mRNAs and microRNA biogenesis by recognizing and interacting with m6A methylation, thereby functioning as a tumor promoter in many malignancies [25, 26, 27]. Moreover, previous studies have reported that IGF2BP2 is implicated in inflammatory response and bone function by stabilizing mRNAs [28, 29]. In OA, Yang et al. showed that the METTL3/IGF2BP2 axis promoted the stability and expression of STAT1, which then elevated ADAMTS12 expression by binding to the promoter region of ADAMTS12, thereby facilitating OA progression [30]. CDH11, known as a transmembrane protein, is a member of the calmodulin family and is broadly expressed in multiple cell types; it is a pivotal regulator of a diverse set of cellular events, such as ECM synthesis, cellular adhesion, proliferation, and smooth muscle cell contraction [31]. CDH11 has been found to be implicated in the development of various malignancies, such as gastric cancer [32], osteosarcoma [33], and breast cancer [34]. In this work, both the overexpression of IGF2BP2 or CDH11 enhanced IL‐1β‐induced apoptosis, inflammation, and ECM degradation in chondrocytes. Moreover, the overexpression of IGF2BP2 or CDH11 could reverse the protective effects of circ_0006646 deficiency on chondrocytes under IL‐1β treatment. Additionally, CDH11 is frequently methylated in certain types of tumors, and it was reported that YTHDF1 translationally promoted CDH11 expression by recognizing m6A‐enriched sites of its transcript, thus promoting the osteolytic bone metastasis of breast cancer [35]. Therefore, IGF2BP2 may stabilize CDH11 by reading the m6A sites of CDH11 and then affect OA progression, while the mechanism by which circ_0006646 upregulates IGF2BP2 still needs further study.

## 5 Conclusions

Our study showed that the abnormally high expression of circ_0006646 might be associated with OA progression. Circ_0006646 promoted IL‐1β‐induced chondrocyte dysfunction by increasing CDH11 in an IGF2BP2‐dependent manner. This study provided a novel insight into the therapy of OA.

## Conflicts of Interest

The authors declare no conflicts of interest.

## Supporting information


**Figure S1.** The selection of IL‐1β concentration for cell models. (A) Chondrocytes were treated with increasing doses of IL‐1β (0, 1, 5, 10, or 20 ng/mL) for 24 h, and circ_0006646 expression was detected by qRT‐PCR. (B) Chondrocytes were treated with 10 ng/mL for 0, 6, 12, 24, or 48 h, and circ_0006646 expression was detected by qRT‐PCR. *p < 0.05.

**Figure S2.** IGF2BP2 regulated cell proliferation, apoptosis, inflammation, and ECM degradation in IL‐1β‐treated chondrocytes. (A) Western blotting was carried out to measure the knockdown efficiency of sh‐IGF2BP2 in chondrocytes. (B‐H) Chondrocytes were divided into three groups: sh‐NC, IL‐1β + sh‐NC, and IL‐1β + sh‐ IGF2BP2. (B‐C) CCK‐8 and EdU assays were performed to detect cell proliferation. (D) Western blot was conducted to detect the protein expression of PCNA. (E) Flow cytometry was conducted to detect cell apoptosis. (F) Western blot was conducted to detect the expression of apoptosis proteins Bcl‐2, Bax, and Cleaved PARP. (G) ELISA was carried out to detect the levels of TNF‐α and IL‐1β inflammatory factors. (H) Western blot was implemented to detect the expression of ECM proteins (Collagen II, Aggrecan, MMP13, and ADAMTS5). *p < 0.05.

**Table S1.** The clinical characteristics of OA patients and Controls.

**Table S2.** Association between the serum levels of circ_0006646 and clinical data for OA patients.

## Data Availability

The data that support the findings of this study are available from the corresponding author upon reasonable request.
