# Supplementary figures and images for: Circ_0006646 Promotes the Progression of Osteoarthritis via Upregulating CDH11 Expression in an IGF2BP2‐Dependent Manner
Source: Kaohsiung J Med Sci. 2025 May 19;41(8):e70031. doi: 10.1002/kjm2.70031 (PMC12407339; doi:10.1002/kjm2.70031)

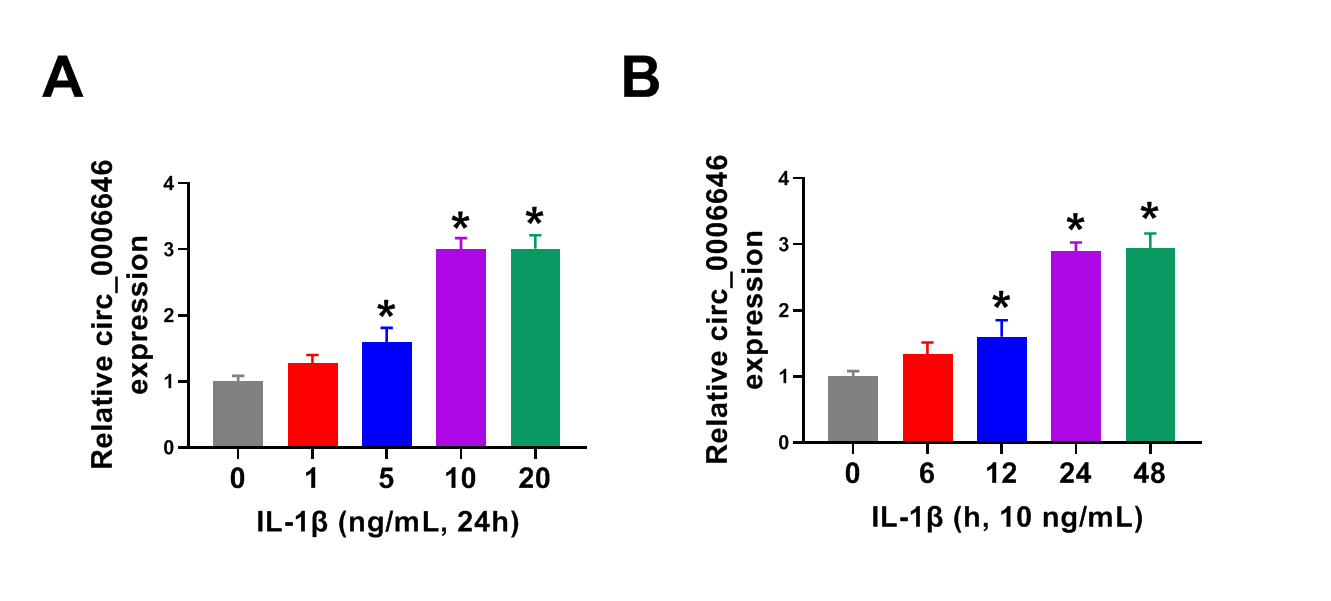

Supplement: Supplementary file 1 — Figure S1. The selection of IL‐1β concentration for cell models. (A) Chondrocytes were treated with increasing doses of IL‐1β (0, 1, 5, 10, or 20 ng/mL) for 24 h, and circ_0006646 expression was detected by qRT‐PCR. (B) Chondrocytes were treated with 10 ng/mL for 0, 6, 12, 24, or 48 h, and circ_0006646 expression was detected by qRT‐PCR. *p < 0.05. [file KJM2-41-e70031-s003.tif]

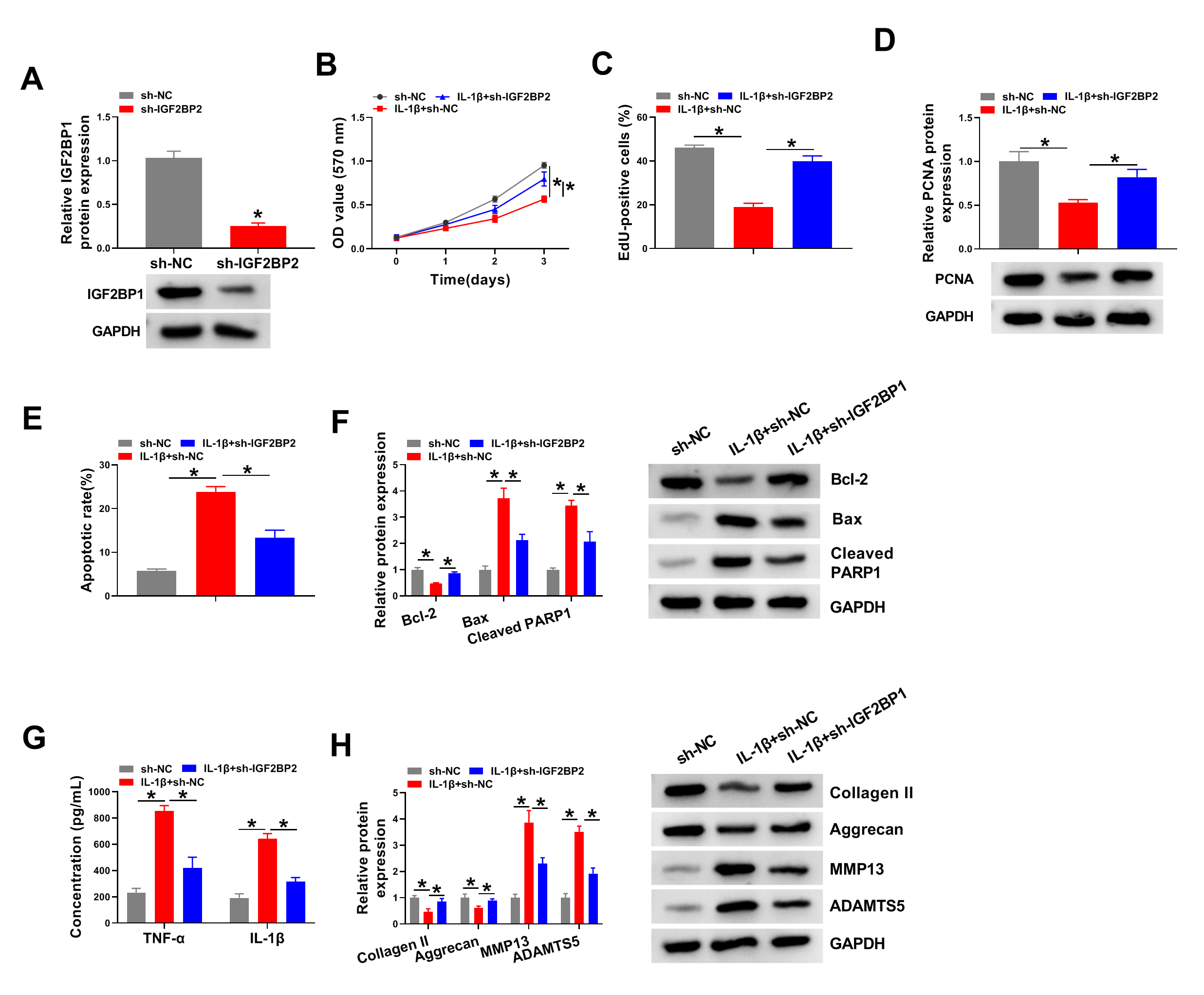

Supplement: Supplementary file 2 — Figure S2. IGF2BP2 regulated cell proliferation, apoptosis, inflammation, and ECM degradation in IL‐1β‐treated chondrocytes. (A) Western blotting was carried out to measure the knockdown efficiency of sh‐IGF2BP2 in chondrocytes. (B‐H) Chondrocytes were divided into three groups: sh‐NC, IL‐1β + sh‐NC, and IL‐1β + sh‐ IGF2BP2. (B‐C) CCK‐8 and EdU assays were performed to detect cell proliferation. (D) Western blot was conducted to detect the protein expression of PCNA. (E) Flow cytometry was conducted to detect cell apoptosis. (F) Western blot was conducted to detect the expression of apoptosis proteins Bcl‐2, Bax, and Cleaved PARP. (G) ELISA was carried out to detect the levels of TNF‐α and IL‐1β inflammatory factors. (H) Western blot was implemented to detect the expression of ECM proteins (Collagen II, Aggrecan, MMP13, and ADAMTS5). *p < 0.05. [file KJM2-41-e70031-s004.tif]
