# Supplementary material for: Circ_0006646 Promotes the Progression of Osteoarthritis via Upregulating CDH11 Expression in an IGF2BP2‐Dependent Manner
Source: Kaohsiung J Med Sci. 2025 May 19;41(8):e70031. doi: 10.1002/kjm2.70031 (PMC12407339; doi:10.1002/kjm2.70031)
Supplement: Supplementary file 3 — Table S1. The clinical characteristics of OA patients and Controls. [file KJM2-41-e70031-s002.docx]

**Table S1. The clinical characteristics of the patients with** **OA and Controls.**

|  | Control (n=21) | OA (n=30) |
| --- | --- | --- |
| Gender |  |  |
| Males | 9(42.86%) | 17(56.67%) |
| Females | 12(57.14%) | 13(43.33%) |
| Age (years) | 54.52±4.82 | 54.23±6.31 |
| Body mass index (kg/m^2^ ) | 24.21±2.29 | 24.85±2.57 |
| Kellgren-Lawrence grade |  |  |
| 2 | / | 15(50.00%) |
| 3 | / | 8(26.67%) |
| 4 | / | 7(23.33%) |
