# Supplementary material for: Circ_0006646 Promotes the Progression of Osteoarthritis via Upregulating CDH11 Expression in an IGF2BP2‐Dependent Manner
Source: Kaohsiung J Med Sci. 2025 May 19;41(8):e70031. doi: 10.1002/kjm2.70031 (PMC12407339; doi:10.1002/kjm2.70031)
Supplement: Supplementary file 4 — Table S2. Association between the serum levels of circ_0006646 and clinical data for OA patients. [file KJM2-41-e70031-s001.docx]

**Table S2. Association between the serum levels of circ_0006646 and clinical data for OA patients**

**.**

|  | N=30 | High (n=15) | Low (n=15) | *P* value |
| --- | --- | --- | --- | --- |
| Gender |  |  |  | 0.713 |
| Males | 17 | 9 | 8 |  |
| Females | 13 | 6 | 7 |  |
| Age (years) |  |  |  | 0.143 |
| <55 | 16 | 6 | 10 |  |
| ≥55 | 14 | 9 | 5 |  |
| Body mass index (kg/m^2^ ) |  |  |  | 0.542 |
| <24 | 12 | 5 | 7 |  |
| 24≤BMI<28 | 13 | 8 | 5 |  |
| ≥28 | 5 | 2 | 3 |  |
| Kellgren-Lawrence grade |  |  |  | 0.149 |
| 2 | 15 | 5 | 10 |  |
| 3 | 8 | 6 | 2 |  |
| 4 | 7 | 4 | 3 |  |
